# ACPPfel: Explainable deep ensemble learning for anticancer peptides prediction based on feature optimization

**DOI:** 10.3389/fgene.2024.1352504

**Published:** 2024-02-29

**Authors:** Mingyou Liu, Tao Wu, Xue Li, Yingxue Zhu, Sen Chen, Jian Huang, Fengfeng Zhou, Hongmei Liu

**Affiliations:** ^1^ School of Biology and Engineering (School of Health Medicine Modern Industry), Guizhou Medical University, Guiyang, China; ^2^ Engineering Research Center of Health Medicine Biotechnology of Guizhou Province, Guizhou Medical University, Guiyang, China; ^3^ School of Life Science and Technology, University of Electronic Science and Technology, Chengdu, China; ^4^ School of Healthcare Technology, Chengdu Neusoft University, Chengdu, China; ^5^ College of Computer Science and Technology, and Key Laboratory of Symbolic Computation and Knowledge Engineering of Ministry of Education, Jilin University, Changchun, China

**Keywords:** anticancer peptides (ACPs), deep convolutional neural network (DCNN), ensemble learning, feature optimization, explainable learning

## Abstract

**Background**: Cancer is a significant global health problem that continues to cause a high number of deaths worldwide. Traditional cancer treatments often come with risks that can compromise the functionality of vital organs. As a potential alternative to these conventional therapies, Anticancer peptides (ACPs) have garnered attention for their small size, high specificity, and reduced toxicity, making them as a promising option for cancer treatments.

**Methods**: However, the process of identifying effective ACPs through wet-lab screening experiments is time-consuming and requires a lot of labor. To overcome this challenge, a deep ensemble learning method is constructed to predict anticancer peptides (ACPs) in this study. To evaluate the reliability of the framework, four different datasets are used in this study for training and testing. During the training process of the model, integration of feature selection methods, feature dimensionality reduction measures, and optimization of the deep ensemble model are carried out. Finally, we explored the interpretability of features that affected the final prediction results and built a web server platform to facilitate anticancer peptides prediction, which can be used by all researchers for further studies. This web server can be accessed at http://lmylab.online:5001/.

**Results**: The result of this study achieves an accuracy rate of 98.53% and an AUC (Area under Curve) value of 0.9972 on the ACPfel dataset, it has improvements on other datasets as well.

## 1 Introduction

The BLOBOCAN 2020 statistics drew a grim picture of the global cancer burden with 19.29 million new diagnosis and 9.95 million cancer-related fatalities (Sung et al., 2021). In the following year, both China and the United States reported 4.82 million and 2.37 million new cases, respectively. The alarming figures from 2022 indicated over 19.3 million new cases globally. The continual prevalence posed the urgent quest for potential anticancer drugs (Chhikara and Parang, 2023).

Existing therapeutic strategies for cancer encompasses surgical interventions, radiation, chemotherapy, and immunotherapy. But they frequently present a myriad of complications (Berger et al., 2023). These include infections, pronounced immunosuppression, bleeding, and other severe side effects that jeopardize patient wellbeing (Timmons and Hewage, 2021). In this context, anticancer peptides (ACPs) emerge as a promising alternative since it typically consists of 10–60 amino acids and derived from the biological immune system. ACPs are characterized by their ability to impede tumor progression and with a diminished potential for drug resistance (Turánek et al., 2015; Xie et al., 2020).

The rapid advancements in sequencing technology coupled with the proliferation of high-throughput peptide datasets have ignited interest in machine learning and deep learning for peptide identification. Agrawal et al. employed the ETree learning paradigm for ACPs prediction (Agrawal et al., 2020). Another tool iAMP-2L centered its predictions on antibiotic peptides, diverging from a sole focus on anticancer variants (Xiao et al., 2013). AMPfun (Chung et al., 2020) and xDeep-AcPEP (Chen J. et al., 2021) have Using deep learning methods to predict the multifaceted functionalities of peptides, while Alsanea et al. employed ensemble techniques for ACPs prediction (Alsanea et al., 2022). Advanced models like ME-ACP (Feng et al., 2021) and ACP-DA (Chen X. G. et al., 2021) which successfully integrated neural network architectures and data balancing techniques. Equally impressive is the approach taken by Lv et al. that married the light gradient booster with deep representation learning algorithms (Lv et al., 2021a). ENNAACT synergized BiLSTM, CNN, and LightGBM algorithms to achieve the ACPs prediction accuracy 78.95% (Yuan et al., 2023).

Inspired by the research of above scholars, we developed a deep convolutional neural network (DCNN) algorithm model that integrates feature selection, feature reduction, regularization, dropout, and other optimization methods. Based on this foundation, we introduce the idea of integrated algorithms and ensemble 10 machine learning methods as the final cancer peptides prediction model. Furthermore, our model interpretation endeavors spotlight pivotal feature combinations instrumental in shaping the classification outcomes using the technique in Li, Z’s research (Li, 2022).

## 2 Materials and methods

### 2.1 Datasets

To construct a main research dataset, we opted for the most recent anticancer peptides from DBAASP (Yi et al., 2019). We specifically chose peptide sequences designed to target cancer from the database, excluding duplicates and sequences with a length less than 5. Ultimately, we acquired 2,377 anticancer peptide sequences for the positive dataset. Additionally, we selected 2,377 peptide sequences without antimicrobial activity for the negative dataset. Consequently, we assembled a dataset named ACPfel, comprising 4,754 peptide sequences as the main dataset.

In contrast, this paper utilized the same datasets as the benchmark studies by Lv et al. (Lv et al., 2021a) and Yuan et al. (Yuan et al., 2023). And introduced the ACP740 datasets constructed in ACP-DL (Pirtskhalava et al., 2021) The ACP740 dataset comprised 376 ACPs and 374 non-ACPs. Additionally, we sourced the ACPs data from the CancerPPD (Atul et al., 2015). From this database, we selected a main dataset of 688 ACPs and an equal number of non-ACPs to create the training dataset. The remaining 171 samples from each category were chosen to form the test dataset. Furthermore, we introduced the CancerPPD alternative dataset, which consisted of 970 experimentally validated ACPs and an equal number of non-ACPs. Within this alternative dataset, the training set was constructed using 776 samples from each class. The remaining 194 ACPs and 194 non-ACPs were set aside to serve as the testing dataset. All the training, validation, and testing datasets used are presented in Table 1.

**TABLE 1:** The benchmark research datasets of this paper.

Datasets	Training datasets	Test datasets	In total
ACP740	629	111	740
CancerPPD main dataset	1,376	342	1718
CancerPPD alternative dataset	1,552	388	1940
ACPfel dataset	3,327	1,427	4,754

### 2.2 Sequence encoding

An ACP sequence is denoted as *P* = *R*_1_ *R*_2_…*R*_*L*_, where *R*_*i*_ represents the *i* ^th^ amino acid (AA) and *L* is the length of this ACP (Liu M. et al., 2023). Then, in the process, every individual instance of AA (which represents a specific amino acid in a protein sequence) is converted into a binary code using the encoding strategy described in detail in Table 2. This binary encoding strategy assigns a unique binary sequence to each AA, enabling easy representation and manipulation of the amino acid sequences in a binary format. The encoding process involves converting the properties or characteristics of each AA into binary digits, which are then combined to form a binary representation specific to that AA. This binary representation serves as a digital counterpart that can be easily analyzed, compared, and processed in various computational tasks such as sequence alignment, protein folding prediction, or machine learning algorithms. as shown in Table 2.

**TABLE 2:** Encoding strategy of the twenty amino acids (AA).

AA	Encoding	AA	Encoding	AA	Encoding	AA	Encoding
A	00,001	G	00,110	M	01,011	S	10,000
C	00,010	H	00,111	N	01,100	T	10,001
D	00,011	I	01,000	P	01,101	V	10,010
E	00,100	K	01,001	Q	01,110	W	10,011
F	00,101	L	01,010	R	01,111	Y	10,100

We utilized the 5-bit binary encoding instead of the standard 20-bit one-hot method to avoid generating many sparse matrices. During the feature extraction process, we employed BiLSTM to capture the contextual relationships between amino acids. By setting the sequence padding maxlen to 512 in data preprocessing, we enhanced efficiency, as using the traditional 20-bit one-hot encoding would have led to longer vector lengths, demanding more memory, and slowing down training.

### 2.3 Data preprocessing

In the initial phase of preprocessing, we employed the pad sequence function to convert variable-length peptides into a consistent length of 512 by padding. This approach enhances the consistency and comparability of the input data, and ensures the peptides share a uniform length. Then, using the random forest algorithm (Biau, 2012) to extract the features of the training and test sets separately, where the training set serves as the training and learning data for the deep convolutional neural network (DCNN), and the cross-validation is introduced during the training process, building a feature extraction model through k-fold cross-validation, and evaluating the training performance.

Then, extract the features of the intermediate layer of the deep convolutional neural network, and then perform PCA dimensionality reduction on the standardized features. PCA is a renowned method to diminish the dimensionality of a dataset while preserving the major share of the intrinsic variability (Bro and Smilde, 2014). This study uses the principal component analysis (PCA) technique to enrich the peptide encoded feature space and proposes the PCA-enriched ensemble learning framework ACPPfel for the ACPs prediction task. The PCA-based dimensionality-reduced training set features reflect the transformation information of the feature space. These are then dispatched to 10 classification algorithms for rigorous training and evaluation.

### 2.4 Model construction

The entire process of constructing the model involved several steps. Firstly, encoded the anticancer peptide sequences, and then selected the features based on the encoded data using the random forest algorithm. The selected features were subsequently used as the training and learning features for the deep convolution neural network (DCNN). During the training process, the bidirectional long short-term memory (BiLSTM) (Zhang et al., 2022) technique was incorporated to extract contextual information from the features.

**FIGURE 1:**
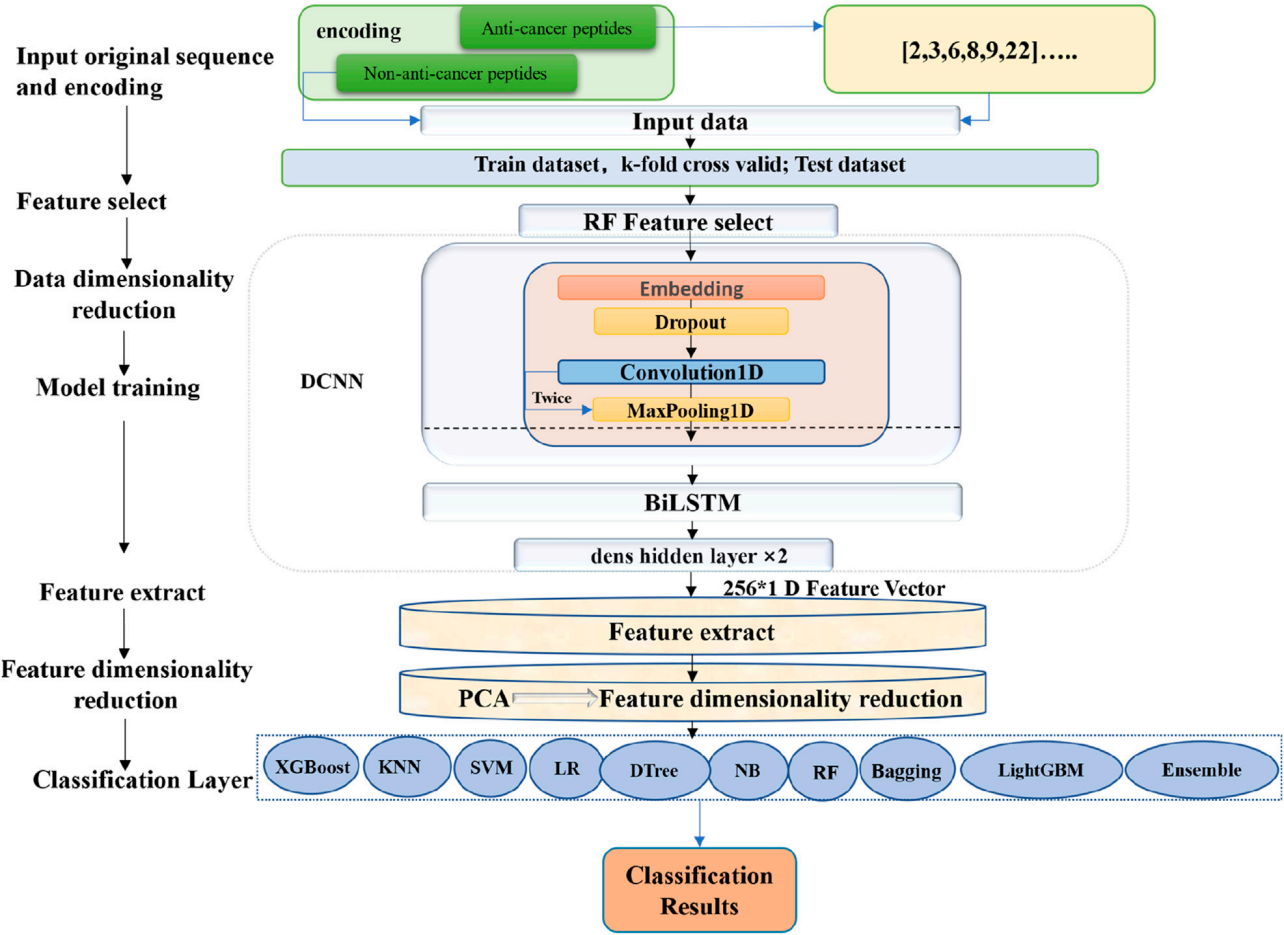
The overall construction process of the ACPPfel model.

After the DCNN training, the feature information from the first layer of the fully connected network was extracted and utilized as input for the subsequent training of the ensemble model. Prior to training the ensemble model, the RobustScaler method in scaler was applied to standardize the data. The processed data were then fed into the PCA algorithm for dimensionality reduction, enriching the information within the feature vectors. The data after dimensionality reduction were subsequently used as input for the ensemble learning algorithm, facilitating the training process, and ultimately leading to the construction of the final predictive model for anticancer peptides. It is worth mentioning that the RobustScaler (Reddy et al., 2021) method is a robust approach for scaling numerical features, providing reliable and accurate results across various machine learning tasks. Additionally, the PCA algorithm was also employed to reduce dimensionality while preserving valuable information in the feature vectors. Finally, the ensemble classifier was built based on ten classifiers. The overall training workflow is shown in Figure 1.

The ACPPfel framework was implemented using Python programming language version 3.9.18. We evaluated ten binary classifiers using the following packages, including keras version 2.8.0, tensorflow version 2.8.0, joblib version 1.1.0, scikit-learn version 1.0.2, xgboost version 1.6.0, and lightgbm version 3.3.5.

1) Support Vector Machine (SVM). SVM has been widely used for both classification and regression tasks (Boopathi et al., 2019). This study used SVM as a binary classifier by the following parameter choices: kernel = ‘poly’, C = 5, gamma = 0.2, degree = 3, coef0 = 0.8, and tol = 1e-3.
2) Random Forest (RF). RF is an ensemble learning method based on multiple decision trees and determines the class label of a sample by the ensembled results of the individual trees. The parameters were set to n_estimators = 10, random_state = 35, criterion = 'entropy’, and max_depth = 50.
3) XGBoost. It is the abbreviation of extreme gradient boosting algorithm with very fast training speed (Chen et al., 2015). The key parameters were max_depth = 50, n_estimators = 100, learning_rate = 0.1, colsample_bytre = 0.7, gamma = 0, reg_alpha = 4, objective = ‘binary: logistic’, eta = 0.3, and subsample = 0.8.
4) K-Nearest Neighbors (KNN). KNN functions by classifying a sample based on its similarity to the neighboring data points and demines a sample’s class label by the majority one of the k neighbors of this query sample (Xing and Bei, 2019). Parameters were set to n_neighbors = 2, p = 1, and metric = ‘euclidean’.
5) Gaussian Naïve Bayes (GNB). This probabilistic classifier roots in Bayes’s theorem, and operates under the assumption of feature independence (Kamel et al., 2019). The var_smoothing parameter was tuned to 1e-05.
6) Logistic Regression (LG). LG models the relationship between a binary dependent variable and one/more independent variables (Shipe et al., 2019). Parameters were adjusted to random_state = 1,000, max_iter = 128, tol = 10, penalty = ‘l2’, and solver = ‘sag’.
7) Decision Tree Classifier (DTree). DTree employs tree-like decision structures, and aims to predict target variables based on the learned decision rules (Yoo et al., 2020). Parameters were criterion = ‘entropy’, random_state = 1, and max_depth = None.
8) Bagging. The Bagging classifier uses the bagging strategy to train on different training data subsets to enhance model accuracy and stability (Sandag, 2020). Key parameters included: criterion = ‘entropy’, random_state = 1, max_depth = None, base_estimator set to the ‘Decision Tree Classifier’, n_estimators = 50, max_samples = 1.0, max_features = 1.0, bootstrap = True, bootstrap_features = False, n_jobs = 1, and another random_state = 1.
9) LightGBM. It is a gradient-boosting framework that uses tree-based algorithms for classification (Yuan et al., 2023). It is optimized for memory efficiency and speed. During training, we employed GridSearchCV for parameter tuning. The ‘num_leaves’ was set to 31, with ‘learn-ing_rate’ and ‘n_estimators’ values fine-tuned in ranges [0.01, 0.1, 1] and [20, 40, 80, 100], respectively. Optimal values were ‘learning_rate’ = 0.01 and ‘n_estimators’ = 80.
10) Ensemble Learning. Our ACPs prediction model utilized the stacking classification technique and comprised a two-step process: constructing primary estimators and integrate them into a holistic estimator (Dong et al., 2020). Five classifiers, namely, RF, XGBoost, LightGBM, DTree, and Bagging, formed the estimators. These classifiers retained the parameter configurations from their individual model descriptions. Finally, the Stacking Classifier technology was harnessed to produce ACPs predictions.

### 2.5 Model explanation

We incorporated the SHAP (Shapley Additive explanation) model (Lundberg and Lee, 2017) to rank the influential features within logistic regression and discern the specific feature combinations with the most profound influence on the ultimate classification outcomes. SHAP is a robust methodology designed to elucidate the results made by prediction models. It aims to calculate the Shapley value as the quantifiable measure of each feature’s contribution towards a given prediction model. This empowers users with explain ability of each feature in the prediction model and has been adopted across diverse areas, including biomedical sciences and healthcare.

SHAP generates locally additive feature attribution as $\left.\hat{y_{i}}={shap}_{0}+{shap(X}_{1i})+{shap(X}_{2i})+\ldots +{shap(X}_{pi}\right)$ , where $\hat{y_{i}}$ is the model prediction value of the observation *i*, ${shap}_{0}$ = $E\left(\hat{y_{i}}\right)$ is the mean prediction across all observations, and $\left.{shap(X}_{ji}\right)$ refers to the SHAP value of the *j* ^th^ feature for observation i , which represents the marginal contribution of the feature to the prediction (Li, 2022).

### 2.6 Model evaluation

The four metrics are used as principal indicators to calculate the performance metrics of an ACP prediction model. True positives represent the positive samples which are correctly identified, and TP is the number of true positives. True negatives denote the correctly classified negative samples, and the number of such samples is TN. False positives and false negatives refer to the samples that are incorrectly tagged as positives and negatives, respectively. TP and TN denote the numbers of false positives and false negatives. Sensitivity and specificity (Skaik, 2008) are defined as SN = TP/(TP + FN) and SP = TN/(TN + FP), respectively. The accuracy ACC=(TP + TN)/(TP + FN + TN + FP) describes the overall rate of the correctly predicted samples. Matthew’s correlation coefficient (MCC) offers a quality measure of a binary classification model and is defined as $MCC=\left(\text{TN}\times \text{TP}-\text{FP}\times \text{FN}\right)/\sqrt{\left(\text{TP}+\text{FP}\right)\left(\text{TP}+\text{FN}\right)\left(\text{TN}+\text{FP}\right)\left(\text{TN}+\text{FN}\right)}$ .

Furthermore, the Receiver Operating Characteristic (ROC) and its accompanying Area Under the ROC Curve (AUC) metrics employed in our evaluation. The ROC curve visually illustrates the trade-offs between the true positive rate (TPR = TP/(TP + FN)) and the false positive rate (FPR = FP/(TN + FP)) over a range of decision thresholds.

The convexity of the ROC curve offers insights into the model’s performance, with a curve skewing towards the top-left corner being indicative of superior predictive capability. The AUC, on the other hand, quantifies the overall performance, with values tending towards 1.0 symbolizing exemplary predictions, while a score around 0.5 is indicative of a model that predicts no better than random chance (Dzisoo et al., 2019).

In essence, this suite of metrics provides a holistic view of our model’s proficiency, ensuring that we capture both its strengths and areas of potential improvement.

## 3 Results

To ensure the stability and comprehensiveness of our delivered model. We constructed a new dataset called ACPfel and utilized three publicly available datasets for training and testing. In the process of model construction, we introduced feature selection mechanisms, Dropout, and regularization methods to overcome the overfitting phenomenon of deep neural networks. The experimental results of our approach were highly encouraging. The model developed exhibited superior performance during independent testing, indicating its robustness and generalizability. To facilitate easy access and utilization of these datasets and the finalized model, we have made them readily available on our web server.

### 3.1 Training dataset model performance

The main dataset, the alternative dataset, ACP740 the ACPfel dataset were utilized during the training process. After data encoding, the dataset is subjected to feature selection using the random forest algorithm. The resulting selected features are then fed into the DCNN for the purpose of learning and training. We used cross-validation methods to evaluate the performance of the training set during the training process.

**FIGURE 2:**
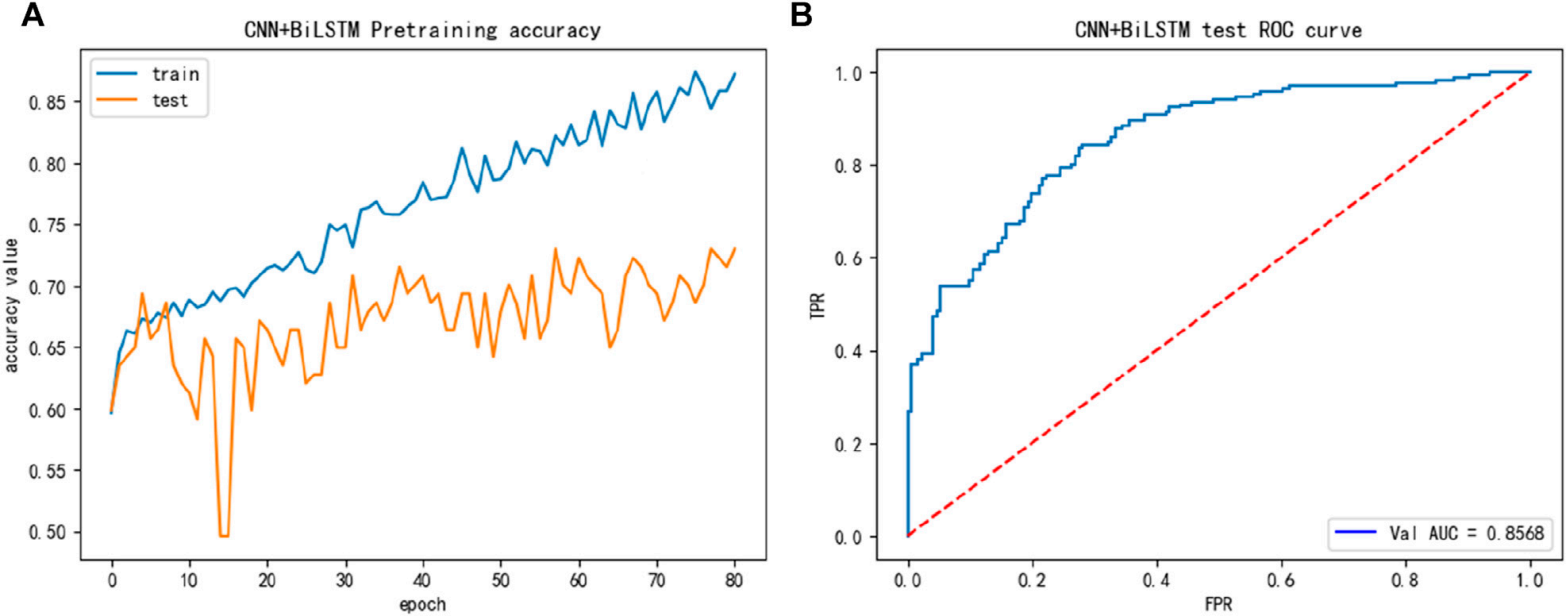
The training performance of main dataset: **(A)** The training accuracy of main dataset; **(B)** The test ROC of the main dataset during training.

The performance of the main dataset training shown in Figure 2, we discovered from Figure 2 that the main dataset exhibited overfitting, and to address this issue, we introduced the dropout layer during the training process and added regularization methods to the fully connected layer, reducing the size of the network, and other measures.

**FIGURE 3:**
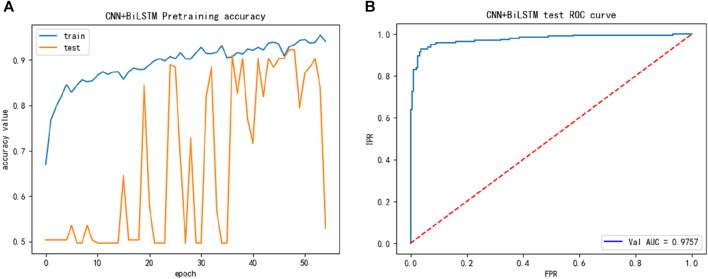
The training performance of alternative dataset: **(A)** The training accuracy of alternative dataset; **(B)** The test ROC of the alternative dataset during training.

To compare studies, we then introduced the alternative dataset, while the performance of the alternative training dataset is shown in Figure 3, we discovered in Figure 3 that the accuracy of the training and test processes reached 90% or higher during training, and its performance was better than that of the main dataset.

**FIGURE 4:**
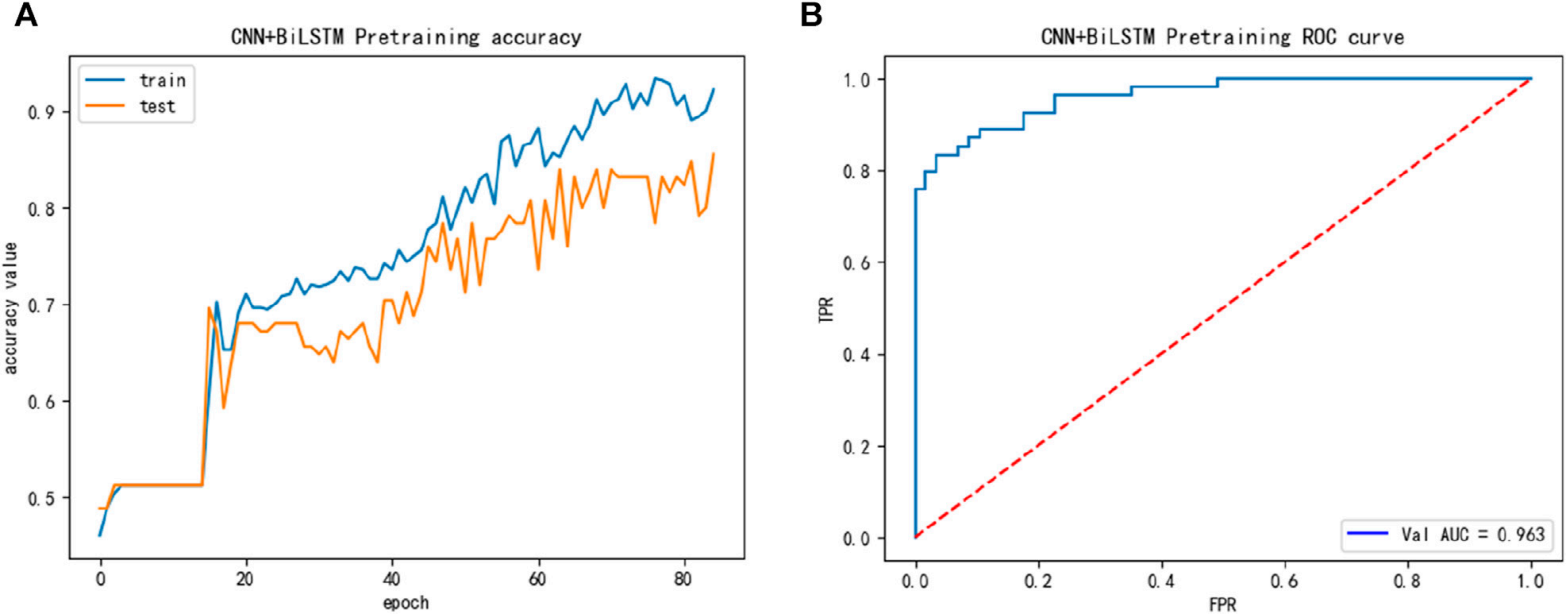
The training performance of ACP740 dataset: **(A)** The training accuracy of ACP740 dataset; **(B)** The test ROC of ACP740 dataset during training.

And then, we introduced the ACP740 dataset, while the performance of the ACP740 training dataset is shown in Figure 4, we discovered in Figure 4 that the ACP740 dataset also exhibited overfitting, but the overall training and cross-validation test performance changed synchronously.

**FIGURE 5:**
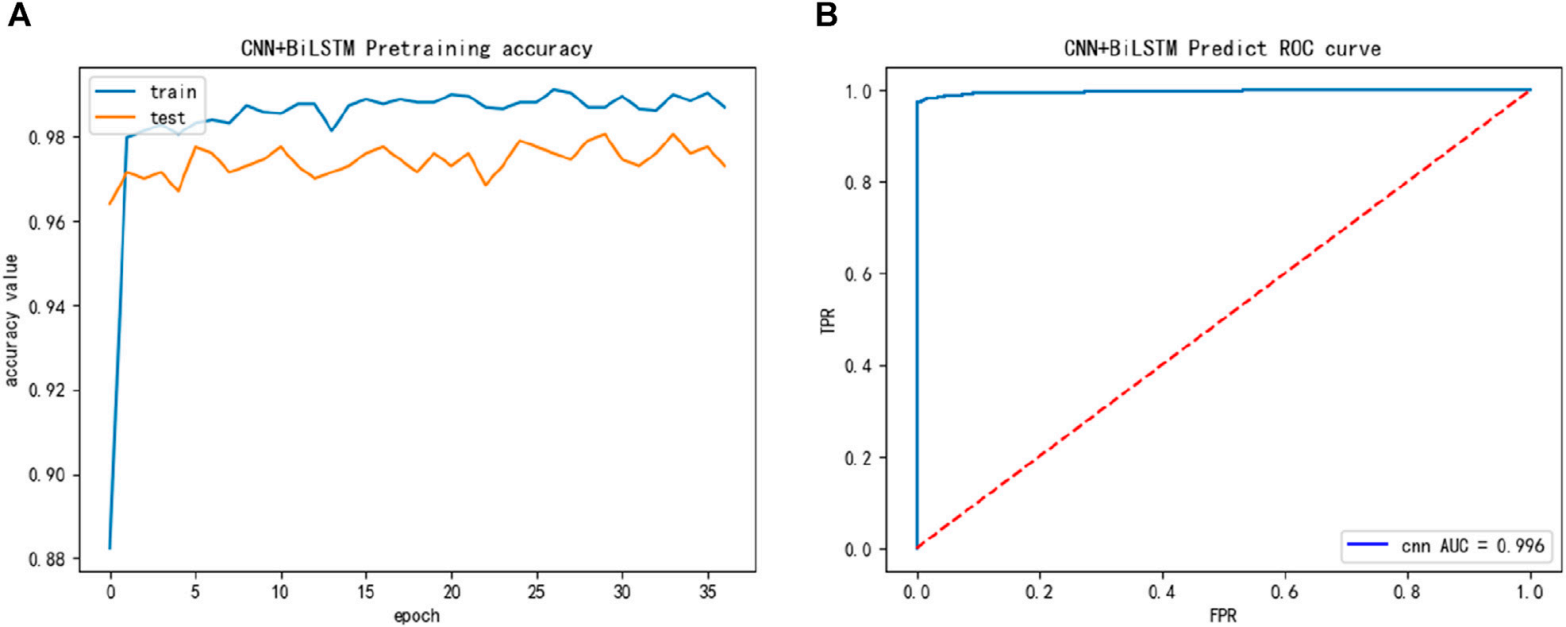
The training performance of ACPfel dataset: **(A)** The training accuracy of ACPfel dataset; **(B)** The test ROC of ACPfel dataset during training.

Finally, we constructed a larger dataset ACPfel, which was collected from the latest databases, containing more recent anticancer peptides, as shown in Figure 5. We found that the performance of the ACPfel training dataset was better during training, with an accuracy of 96% or higher for both training and 5-fold cross-validation, the AUC value was 0.996 and more stable than the other three dataset.

### 3.2 Performance of independent validation

The ACPPfel algorithm is a classification ensemble algorithm designed for prediction anticancer peptides. We constructed the model using the preprocessed training data and then evaluated its performance on two separate independent validation datasets the main one and an alternative one. This approach ensured objectivity in measuring the performance of different classifiers and comparing them objectively. During their extensive comparative research. We found that using the feature selection and PCA algorithm for feature dimensionality reduction improved the performance of the classification ensemble algorithm. This improvement was observed by comparing the performance results presented in Table 3, the best values are highlighted in bold. According to Table 3, the model achieved the highest values for the accuracy reached 78.07%, the sensitivity reached 81.29%, and the specificity reached 78.36%.

**TABLE 3:** The performance of various classification models based on DCNN of the main dataset.

Classification model	MCC	SP	SN	ACC
SVM	0.5440	0.7602	0.7836	0.7719
RF	0.5380	0.7544	0.7836	0.7690
XGBoost	0.5500	0.7544	0.7953	0.7749
KNN	0.4690	0.7661	0.7018	0.7339
GNB	0.5610	**0.7836**	0.7778	0.7807
LG	0.5570	0.7485	0.8070	0.7778
DTREE	0.5010	0.6901	0.8070	0.7485
Bagging	**0.5630**	0.7485	**0.8129**	**0.7807**
LightGBM	0.5500	0.7544	0.7953	0.7749
Ensemble	0.5380	0.7544	0.7953	0.7690

That the bold values indicates the best values.

**FIGURE 6:**
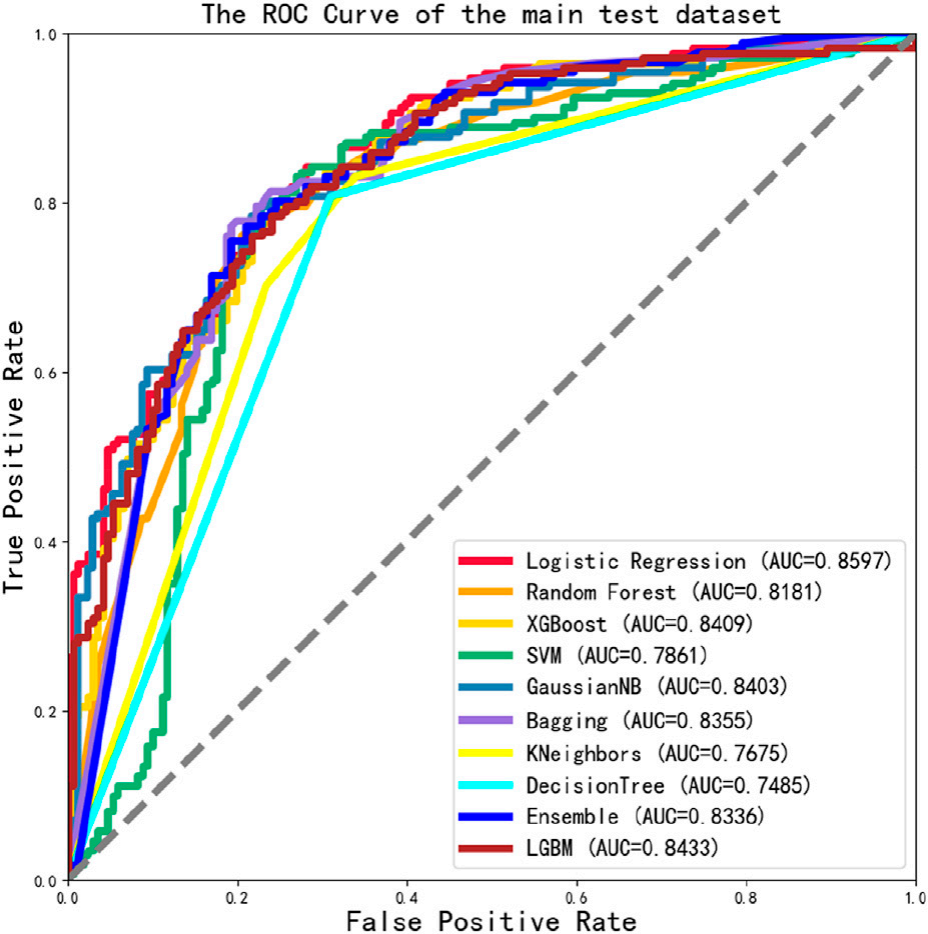
The ROC Curve performance of independent validation dataset of main dataset.

To evaluate the overall performance of the models, the ROC values of 10 different models were assessed simultaneously, as shown in Figure 6, the main dataset highest AUC value is 0.8597.

We then tested the alternative dataset using an independent test set, the best values as shown in Table 4, are highlighted in bold. We found that our model achieved an accuracy (ACC) of 93.56%, with a sensitivity (SN) of 94.33% and a specificity (SP) of 94.33%, the result outperforming the main dataset.

**TABLE 4:** The performance of various classification models based on DCNN of alternative dataset.

Classification model	MCC	SP	SN	ACC
SVM	0.8610	0.9278	0.9330	0.9304
RF	0.8610	0.9330	0.9278	0.9304
XGBoost	0.8660	0.9227	**0.9433**	0.9330
KNN	0.8560	**0.9433**	0.9124	0.9278
GNB	**0.8710**	0.9278	**0.9433**	**0.9356**
LG	**0.8710**	**0.9433**	0.9278	**0.9356**
DTREE	0.8250	0.8969	0.9278	0.9124
Bagging	0.8560	0.9175	0.9381	0.9278
LightGBM	**0.8710**	0.9278	**0.9433**	**0.9356**
Ensemble	0.8660	0.9278	**0.9433**	0.9330

That the bold values indicates the best values.

**FIGURE 7:**
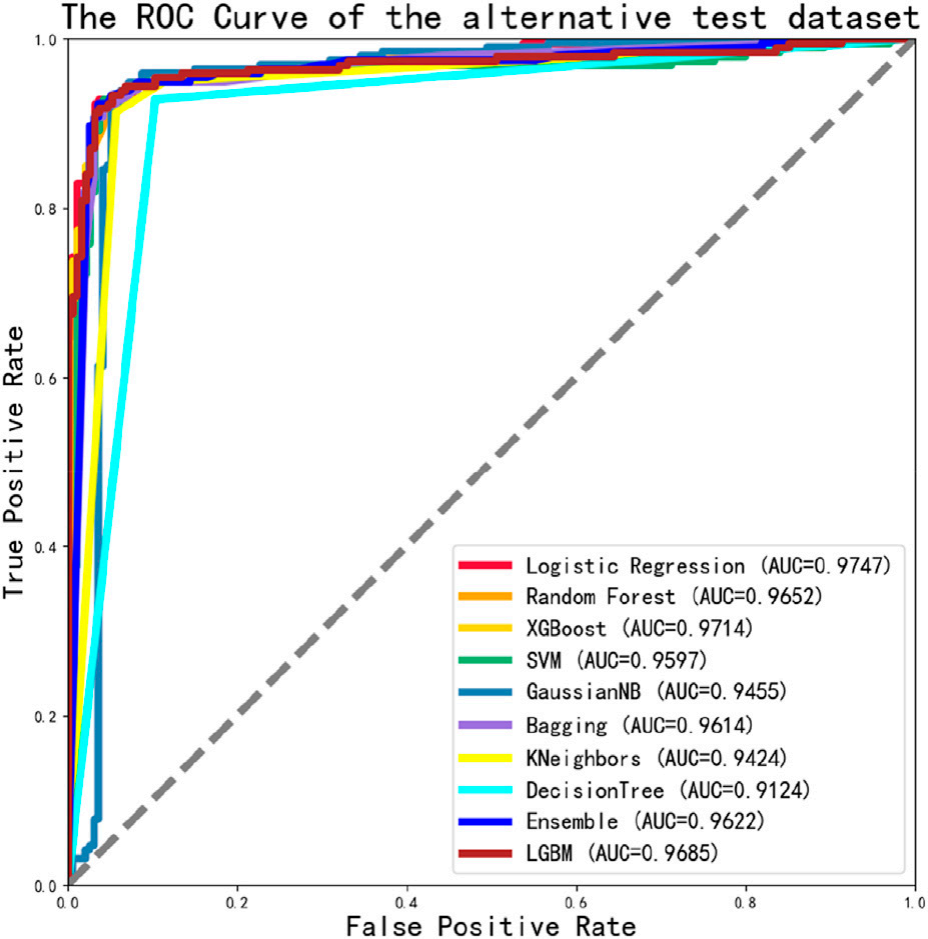
The ROC Curve performance of independent validation dataset of alternative dataset.

We also found that the ROC values of the alternative dataset independent test in 10 classification models were well-performed, all reached a value of 0.9 or higher. And the maximum value was 0.9747, as shown in Figure 7.

To further validate the performance of the model, additional experiments were conducted. We then continued to introduce the ACP740 dataset used in the literature ACP-DL (Yi et al., 2019) for training and testing, with 629 of the data used as the training set, and the 5-fold cross-validation method was used during the training process. The remaining 111 of the data was used as an independent test dataset for the final model testing and validation, the best values as shown in Table 5, are highlighted in bold. We found that the MCC value reached the highest of 0.8380 in Table 5, with SP, SN, and ACC values of 92.98%, 92.59%, and 91.89%, respectively.

**TABLE 5:** The performance of various classification models based on DCNN of the ACP740 dataset.

Classification model	MCC	SP	SN	ACC
SVM	0.7660	0.8772	0.8889	0.8829
RF	0.7660	0.8947	0.8704	0.8829
XGBoost	0.7840	0.9123	0.8704	0.8919
KNN	0.7320	0.9123	0.8148	0.8649
GNB	0.7660	0.8772	0.8889	0.8829
LG	0.7660	0.8947	0.8704	0.8829
DTREE	0.6760	0.8246	0.8519	0.8378
Bagging	**0.8380**	0.9123	**0.9259**	**0.9189**
LightGBM	0.8030	**0.9298**	0.8704	0.9009
Ensemble	0.8030	**0.9298**	0.8704	0.9009

That the bold values indicates the best values.

**FIGURE 8:**
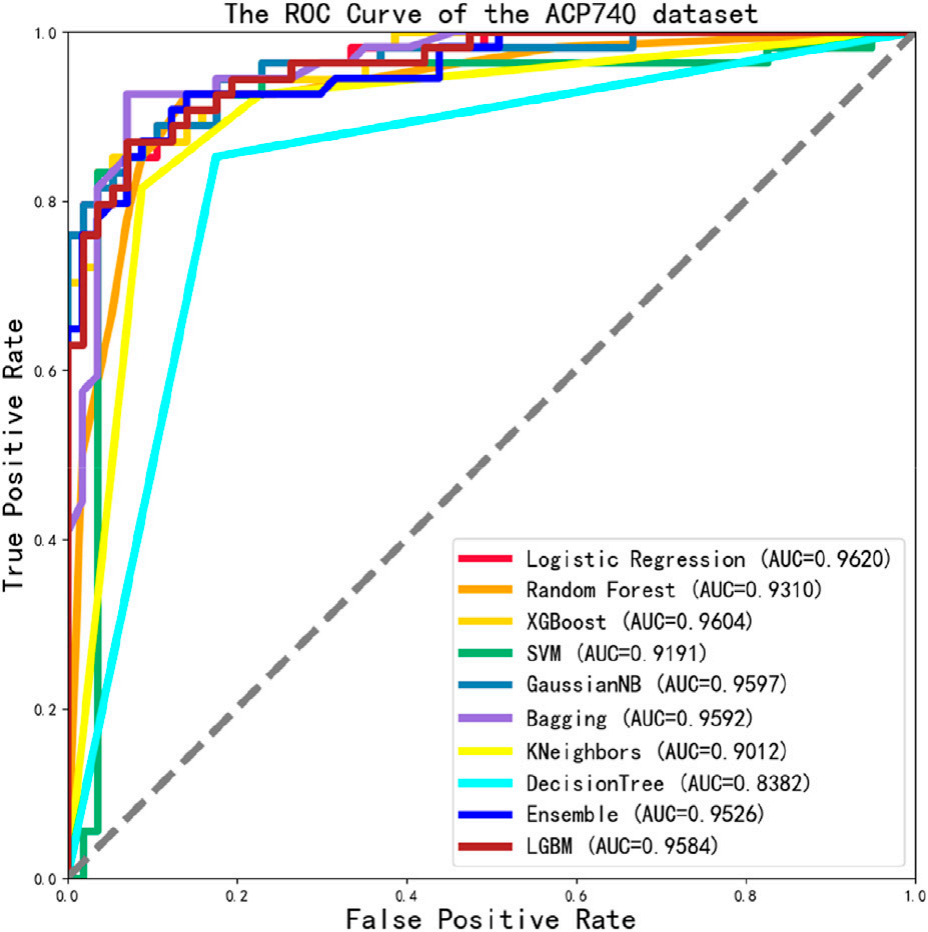
The ROC Curve performance of independent validation dataset of ACP740 dataset.

We tested the ROC values of the ACP740 dataset, as shown in Figure 8. Out of the 10 classification algorithms, 9 of them had values that exceeded 0.9, the LG reaching a maximum value of 0.9620.

We built a training and test dataset called ACPfel that included more recent anticancer peptides from DBAASP (Pirtskhalava et al., 2021), with sequences of length less than 5 removed and duplicate sequences removed. The final dataset consisted of 4,754 sequences, with 3327 of the data used as the training set and cross-validation. The remaining 1427 of the data was used as an independent test dataset, the best values as shown in Table 6, are highlighted in bold.

**TABLE 6:** The performance of various classification models based on DCNN using the ACPfel dataset.

Classification model	MCC	SP	SN	ACC
SVM	0.9680	0.9944	0.9735	0.9839
RF	0.9710	0.9958	0.9749	**0.9853**
XGBoost	0.9670	0.9930	0.9735	0.9832
KNN	0.9670	0.9972	0.9693	0.9832
GNB	**0.9720**	**0.9986**	0.9735	0.9860
LG	0.9660	0.9902	**0.9763**	0.9832
DTREE	0.9550	0.9831	0.9721	0.9776
Bagging	0.9690	0.9944	0.9749	0.9846
LightGBM	0.9690	0.9944	0.9749	0.9846
Ensemble	0.9710	0.9944	0.9749	**0.9853**

That the bold values indicates the best values.

**FIGURE 9:**
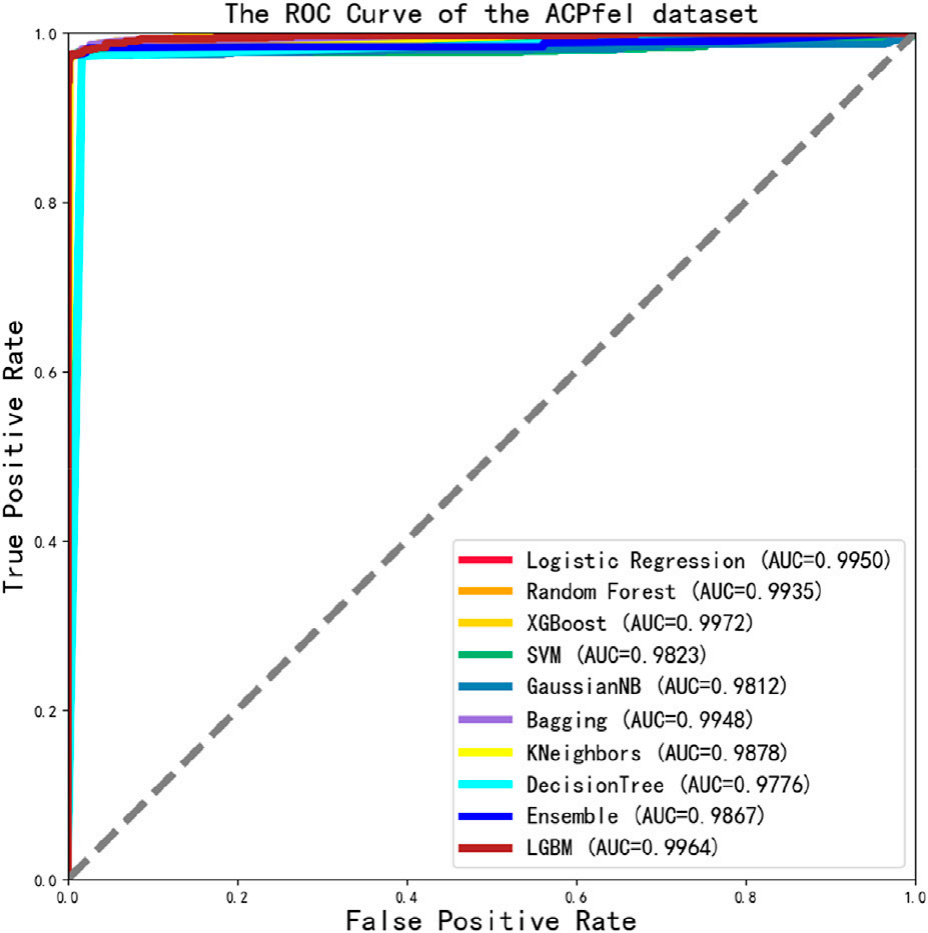
The ROC Curve performance of independent validation dataset of ACPfel dataset.

We found that the performance of 10 classification algorithms in Table 6 was better than others, with the highest MCC value reaching 0.9720 and the highest SP, SN, and ACC values of 99.86%, 97.63%, and 98.53%, and that the final test ROC values exceeded 0.970 or higher, even reaching a maximum value of 0.9972. The performance of ACPfel was better than those of the previous three datasets. The result as shown in Figure 9.

### 3.3 SHAP feature explanation

After predicting anticancer peptides using the logistic regression model, the SHAP algorithm was used to extract the features and rank the feature importance. We used the SHAP Summary Plot method to generate the Summary plot chart, which displayed the Shapley values for each feature of each sample in the past tense. This helped us determine which features were the most important and their impact on the dataset. The *y*-axis represented the feature names, and the *x*-axis represented the influence weight of the Shapley value. The color indicated the feature value (red for high, blue for low). The overlapping points were jittered along the *y*-axis so that we could observe the distribution of Shapley values for each feature, which were sorted according to their importance.

**FIGURE 10:**
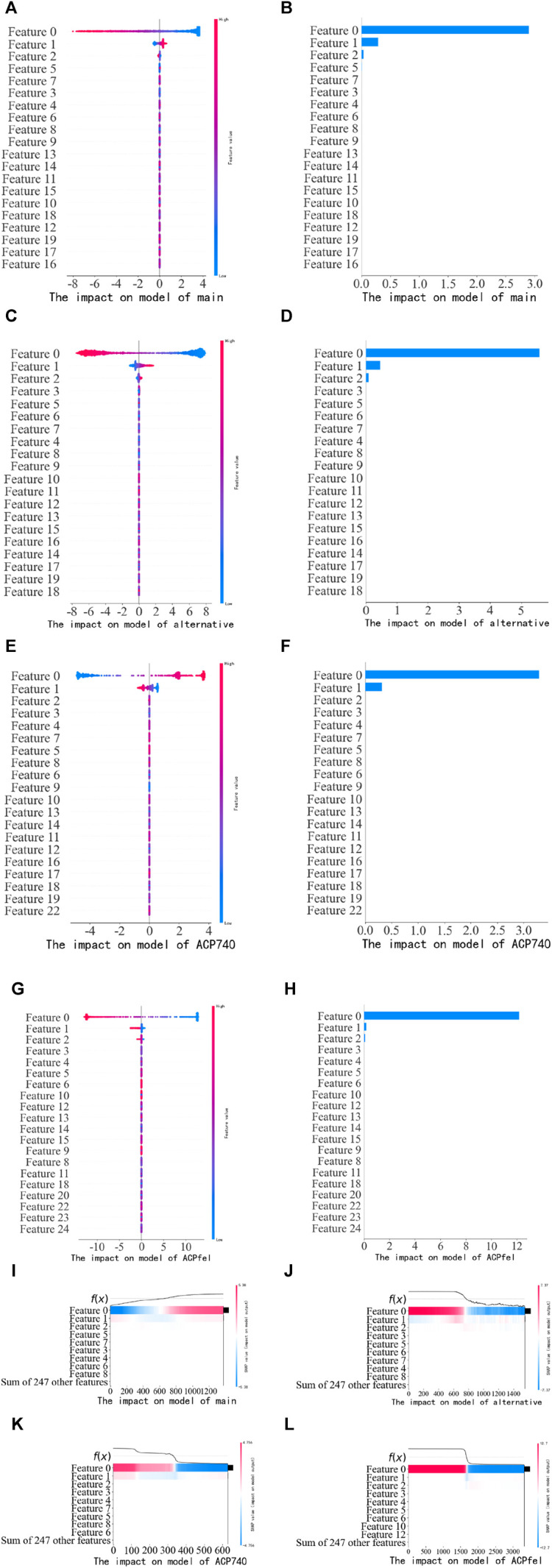
Performance of training dataset on the training model. **(A)** The SHAP values scatter plot of the main dataset training; **(B)** The SHAP values bar chart of the main dataset training; **(C)** The SHAP values scatter plot of the alternative dataset training; **(D)** The SHAP values bar chart of the alternative dataset; **(E)** The SHAP values scatter plot of the ACP740 dataset training; **(F)** The SHAP values bar chart of the ACP740 dataset training; **(G)** The SHAP values scatter plot of the ACPfel dataset training; **(H)** The SHAP values bar chart of the ACPfel dataset training; **(I)** The SHAP values heat map of the main dataset training; **(J)** The SHAP values heat map of the alternative dataset training. **(K)** The SHAP values heat map of the ACP740 dataset training. **(L)** The SHAP values heat map of the ACPfel dataset training.

The results of the experiment are shown in Figure 10. Based on the information presented in Figure 10, it can be observed that when the feature dimensionality is reduced by PCA, feature 0 and feature 1 significantly influence the LG model in the main dataset, as seen in subfigures (A), (B) of Figure 10. Similarly, in the alternative dataset, feature 0, feature 1, feature 2 has the most substantial impact on the LG model, as observed in subfigures (C) and (D) of Figure 10. Similarly, in the ACP740 dataset, feature 0, feature 1 has the most substantial impact on the LG model, as observed in subfigures (E) and (F) of Figure 10, Similarly, in the ACPfel dataset, feature 0, feature 1 has the most substantial impact on the LG model, as observed in subfigures (G) and (H) of Figure 10. This pattern is also visible when examining the SHAP heat map, as shown in subfigures (I), (J), (K) and (L) of Figure 10. To gain insights into which features the models heavily rely on for making their final predictions, it is essential to analyze the feature importance ranking depicted in the figure.

### 3.4 Comparison with the state-of-the-art approaches

There were some previous predictors for ACPs prediction, such as iACP (Chen et al., 2016), PEPred-Suite (Wei et al., 2019), ACPpred-Fuse (Rao et al., 2020a), ACPred-FL (Leyi et al., 2018), ACPred (Schaduangrat et al., 2019), AntiCP (Tyagi et al., 2013), AntiCP_2.0 (Agrawal et al., 2021a), iACP-DRLF (Lv et al., 2021a), they are all test on the main independent validation dataset. To demonstrate the efficacy of our model, we conducted a comparative analysis of its performance with that of previous predictors on the same dataset. The datasets are the same as those used in the benchmark study by (Lv et al., 2021a). The performance of the main dataset shown as in Table 7, Compared to the best model ACP-OPE, our model demonstrated improvements in SP by 1.6%, while the other performances were similar.

**TABLE 7:** The performance of different classification models on main independent validation dataset.

Model	Sens	Spec	Accuracy
ACP-OPE	0.8153	0.7676	0.7895
iACP-DRLF	0.807	0.743	0.775
AntiCP_2.0	0.775	0.734	0.754
AntiCP	1.000	0.120	0.506
ACPred	0.856	0.214	0.535
ACPred-FL	0.671	0.225	0.448
ACPpred-Fuse	0.692	0.686	0.689
PEPred-Suite	0.331	0.738	0.535
iACP	0.779	0.332	0.551
ACPPfel (this paper)	0.8129	0.7836	0.7807

However, in this study, the ACPPfel algorithm was constructed with a highest AUC value of 0.8597 on the main dataset. These results show that the ACPPfel algorithm proposed in this article can better predict the anticancer peptides.

For further verifying the effectiveness of our method, we compared ACPPfel with the existing methods including ACP-DL (Yi et al., 2019), DeepACPpred (Lane and Kahanda, 2021), ACP-MHCNN (Ahmed et al., 2021), GRCI-Net (You et al., 2022), StackACPred (Mishra et al., 2019) on the cross-validation datasets and iACP (Chen et al., 2016), PEPred-Suite (Wei et al., 2019), ACPpred-Fuse (Rao et al., 2020b), ACPred-FL (Wei et al., 2018), ACPred (Schaduangrat et al., 2019), AntiCP (Kumar and Li, 2017), DeepACPpred, AntiCP_2.0 (Agrawal et al., 2021b), iACP-DRLF (Lv et al., 2021b), ME-ACP (Feng et al., 2022) on alternative independent datasets.

From Table 8, we can see that the algorithm model ACPPfel outperforms the current best algorithm in terms of SN, with an increase of 2.63%. The highest performance of ACPPfel in terms of AUC value was 0.9747, compared to the best result of ME-ACP of 0.936, which increased by 3.87%.

**TABLE 8:** The performance of different classification models on alternative independent dataset.

Model	Accuracy	Sens	Spec	MCC
ACP-MHCNN	0.900	0.865	0.933	0.800
iACP-DRLF	0.776	0.784	0.964	0.550
AntiCP_2.0	0.920	0.923	0.918	0.840
AntiCP	0.900	0.897	0.902	0.800
ACPred	0.853	0.871	0.835	0.710
ACPred-FL	0.438	0.602	0.256	−0.15
ACPpred-Fuse	0.789	0.644	0.933	0.600
PEPred-Suite	0.575	0.402	0.747	0.160
iACP	0.776	0.784	0.964	0.550
ACP-DL	0.881	0.860	0.902	0.762
ME-ACP	0.933	0.917	0.948	0.866
ACPPfel (this paper)	0.9356	0.9433	0.9433	0.8710

To make a more objective comparison, we also introduced the ACP740 dataset used in the ACP-DL paper for evaluation, as shown in Table 9. We can see that our proposed algorithm has improved 2.29%, 5.89%, and 4.2% in terms of ACC, SN, and MCC, respectively, and the highest AUC value has been improved to 0.9620.

**TABLE 9:** The performance of different classification models on ACP740 independent dataset.

Model	Accuracy	Sens	Spec	MCC
ACP-MHCNN	0.860	0.889	0.831	0.720
DeepACPpred	0.850	0.853	0.850	0.706
GRCI-Net	0.823	0.836	0.821	0.647
StackACPred	0.845	0.841	0.849	0.705
ACP-DL	0.815	0.826	0.806	0.631
ME-ACP	0.896	0.867	0.922	0.796
ACPPfel (this paper)	0.9189	0.9259	0.9298	0.8380

Finally, we constructed a more larger anticancer peptides dataset and evaluated its performance with this dataset. The highest ACC, SN, and SP values were 98.53%, 97.63%, and 99.86%, respectively, and the AUC value reached 0.9972, as shown in Table 6 and Figure 9, it indicates that all indicators were very well-performed.

### 3.5 Web server interface and functional confirmation

**FIGURE 11:**
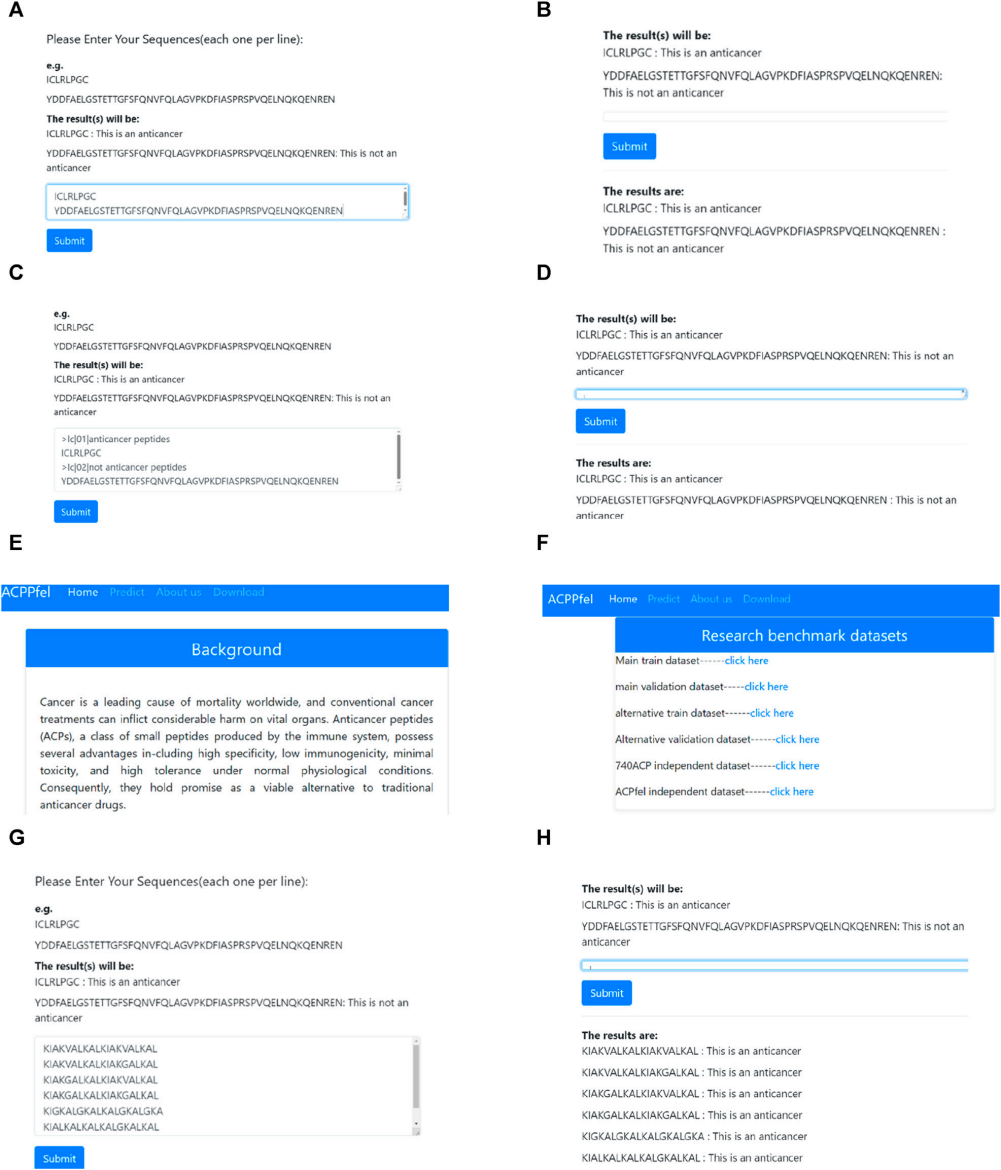
Anticancer Peptides Prediction System. **(A)** Enter the peptides sequence data that need to be predict; **(B)** Submit the peptides sequence that need to be predicted and obtain the prediction results; **(C)** FASTA format data input; **(D)** Submit the FASTA format data peptides sequence and obtain the prediction results; **(E)** Introduction of the study; **(F)** The benchmark dataset of this study. **(G)** Enter the newest six peptides sequence data that need to be predict; **(H)** Submit the newest six peptides sequence that need to be predict and obtain the final prediction results.

By introducing web server technologies such as Flask and HTML (Yang et al., 2021; Zhou et al., 2022), We have developed a web server system for analyzing anticancer peptides data. Users can input the sequence of peptides they want to analyze directly on the webpage and submit it to the analysis system by clicking the “Submit” button. The new peptide sequence is then fed into the ensemble prediction system. If the model’s prediction threshold exceeds 0.5, it indicates that the sequence is an anticancer peptide. Otherwise, it is classified as a non-anticancer peptide. The results of the peptide sequence prediction are displayed at the bottom of the webpage. Figure 11 illustrates the process of the analysis demonstration.

Based on Figure 11, the analysis system can analyze each peptide sequence data, predict whether the sequence is an anticancer peptide, and provide the results at the bottom of the webpage. This article has already been established a web server for anticancer peptide prediction which can be accessed at http://lmylab.online:5001/. The web server not only offers predictions for anticancer peptides but also provides a download link for the benchmark datasets used in the study, allowing users to access them for further research purposes.

To verify the reliability of the model, we downloaded the latest discovered anticancer peptide sequences from the DBAASP database (Pirtskhalava et al., 2021), Through biological experiments, it has been demonstrated that this sequence possesses functions such as anti-Gram+, anti-Gram-, and anticancer (Rončević et al., 2023). Moreover, this sequence is not included in our training dataset. When we performed prediction on this sequence using the web server developed in this study, it was found that our model can accurately predict the latest anticancer peptide sequences, as shown in (G), (H) of Figure 11.

## 4 Discussion

Cancer as a disease caused by pathological changes in cellular division, has become a leading cause of death worldwide. The persistent prevalence of cancer worldwide results in the loss of millions of lives annually. Traditional cancer treatment methods often inflict significant harm on patients. However, Anticancer peptides (ACPs) offer several advantages including high specificity, low immunogenicity, minimal toxicity, and high tolerance under normal physiological conditions. It provides a potential alternative for cancer treatment. Traditional laboratory methods for identifying these peptides are time-consuming, expensive, and inefficient. In contrast, machine learning methods can be used to predict anticancer peptides, requiring only computational resources (Zhou et al., 2023a; Zhou et al., 2023b). This approach offers a more efficient and cost-effective means of identifying potential candidates for anticancer therapy (Liu X. W. et al., 2023; Yang et al., 2022).

During the training of the anticancer peptides prediction model, the deep convolution neural network (DCNN) (Zhou et al., 2023b) model is prone to overfitting issues due to both the limited size of the dataset and the influence of interfering data. To address these concerns, we performed feature extraction prior to training to eliminate interfering data. Additionally, we incorporated techniques such as dropout, Batch Normalization and Regularizers to enhance the simplicity of the network. In future research, we intend to explore more methods to effectively mitigate this problem.

To evaluate the model, we conducted a systematic evaluation and comparison analysis of the final performance of the model with other related studies. Firstly, we compared our algorithm with the best result of ME-ACP (Feng et al., 2022). As shown in Table 8, ACPPfel had a 2.63% higher SN on an alternative independent dataset. The best performance of ACPPfel in terms of AUC value was 0.9747, which was 3.87% higher than that of ME-ACP. We also introduced the ACP740 dataset used in the ACP-DL (Yi et al., 2019) for evaluation. As shown in Table 9, our model has improved 2.29%, 5.89%, and 4.2% in terms of ACC, SN, and MCC, respectively, and the best AUC value has been improved to 0.9620. Finally, we constructed a larger anticancer peptides dataset and evaluated its performance with this dataset. The highest ACC, SN, and SP values were 98.53%, 97.63%, and 99.86%, respectively, and the AUC value reached 0.9972, as shown in Table 6 and Figure 9. ACPPfel has made and optimized based on DCNN using many techniques, including a feature selection algorithm to reduce interference data, BiLSTM to extract context features from the anticancer sequence during training, and the middle layer feature of the fully connected layer as the learning feature for the ensemble algorithm. The entire process is performed in multiple steps of dimensionality reduction, which improves the training speed, and the SHAP algorithm is introduced to backtrack to find the feature combination that affects the result.

In future research, based on the approach mentioned, investigators undertook additional exploratory studies to delve into the intricate biological mechanisms that drive the anticancer activity inherent in peptide sequences. This rigorous investigation involved the inclusion of various biological experiments to not only validate the findings but also unravel the biological significance and interpretability of the observed effects. By thoroughly understanding the underlying biological mechanisms, this research will establish a solid theoretical groundwork that can guide the later stages of developing effective and targeted anticancer peptide drugs. The comprehensive understanding gained from these studies will aid in the identification and design of potential therapeutic peptides with optimal properties, paving the way for more successful drug development efforts in the future.

## 5 Conclusion

In this research project, we have developed a model for predicting anticancer peptides by ten classification algorithms to analyze and identify anticancer peptides data.

To overcome the challenges posed by high feature dimensionality and the presence of irrelevant feature information, we introduced the feature selection and PCA algorithm for dimensionality reduction during the feature extraction process. This approach aimed to mitigate noise interference and enhance the overall performance of the algorithm.

To validate the effectiveness of our proposed algorithm, we utilized the same dataset as the benchmark paper by Lv Z, et al. (Lv et al., 2021a). Independent testing with this dataset demonstrated that our algorithm achieved comparable performance to existing anticancer peptide prediction algorithms in terms of accuracy, sensitivity, MCC, and other evaluation metrics. Furthermore, when compared with state-of-the-art algorithms, our approach exhibited improvements and yielded better results.

In future research endeavors, our objective is to enhance the interpretability of the algorithm from a biological standpoint. Additionally, we aim to verify the functional activities of anticancer peptides through wet laboratory experiments, thereby establishing a comprehensive understanding of their potential applications.

## Data Availability

The datasets presented in this study can be found in online repositories. The names of the repository/repositories and accession number(s) can be found in the article/supplementary material.
